# A Circular

**Published:** 1872-07-15

**Authors:** David Prince

**Affiliations:** Jacksonville, Ill.


### A CIRCULAR.

*To the Medical Profession:* The undersigned was appointed, at the last Annual Meeting of the Illinois State Medical Society, a committee to report at the next Annual Meeting of the Society, upon the subject of Galvano-therapeutics.

The accomplishment of the object of the appointment will be aided by physicians who may send for treatment obstinate cases of scrofulous or cancerous tumors, rigid palsy, and stiffness or deformity arising from rheumatic deposit.

Much interest has lately been excited by the success of the continuous galvanic current, of great quantity and low intensity, in resolving growths and deposits of low vitality.

Neuralgia, depending upon perverted or diminished nutrition of nerves, has proved remediable by this agent.

The instrument now in use for these investigations is one of 32 cells, of platinum and zinc combination, made by Curt W. Meyer, of New York, and one of 100 cells, made by the New York Galvano-faradic Manufacturing Company, furnishing a much greater quantity, without great increase of intensity.

It is hoped that by these appliances, employed in the treatment of a great variety of cases, some progress may be made in determining the real value of galvanism, and the peculiarities which render cases remediable or intractable. Very truly yours,

David Prince.

Jacksonville, Ill., June, 1872.

Primary Medical Institute.—We have received the Second Annual Circular of the St. Paul School for Medical Instruction. The men engaged in this school are reliable, and its objects highly commendable. They are well stated in the following paragraph from the circular:

The object of this school is not to represent, or in any way take the place of a regular *College*, but to prepare students for a better understanding of the lectures which they will hear in the College course, and to drill them more thoroughly in the elementary branches than can be done in the short time allowed by Colleges for instruction, and the arrangement of terms of study will be such that they will not interfere with the winter course of the Chicago Colleges.

Students on arriving in St. Paul will call at once on the Secretary, who will assist them in finding board, etc.

For any further particulars, address

Alex. J. Stone, M.D, *Secretary*,

34 Jackson st., St. Paul, Minn.

